# Proceedings of the Western Dental Association

**Published:** 1860-08

**Authors:** 


### PROCEEDINGS OF THE WESTERN DENTAL ASSOCIATION.

THIS Association held its fifth annual meeting in the city of Springfield, Ills., on the 5th of July. Its sessions were held in the Senate Chamber, and although not largely attended, the meetings were very harmonious and interesting.

The meeting was called to order at 11 o'clock, A. M., by the President, C. W. Spalding, of St. Louis; and in the absence of the regular Secretary, Dr. Bogin, of Chicago, Dr. A. W. French was appointed Secretary *pro tem.*

The Minutes of the preceding annual meeting were read and approved.

After some desultory discussion, the Association passed a resolution of invitation to the dentists of the city and vicinity to participate in the meeting, after which adjourned to meet at 2½ o'clock, P. M.

2½ O'CLOCK, P. M.—The Association met according to adjournment.

The reports of committees were presented and disposed of, after which, proceeded to the election of officers, which resulted as follows:

*President*—Henry Barron.

*Vice Presidents*—A. Blake and A. W. French.

*Recording Secretary*—C. W. Spalding.

*Corresponding Secretary*—S. Dunham.

*Treasurer*—H. E. Peebles.

*Executive Committee*—I. Forbes, W. W. Alport, G. Perkins, H. E. Peebles, and A. W. French.

JULY 6TH, 9 O'CLOCK, A. M.—The Society met pursuant to adjournment, in the Senate Chamber.

The installation of the officers being the first business of the morning, Dr. Spalding, the retiring President, proceeded to deliver an eloquent address upon the topics of Dental Associations and the benefits to be derived from them by their members. He said that it is one of the glories of the dental profession to throw aside old forms and usages, and to strike out in new channels. There is a degree of independent thought and action in the dental profession which does not arise in others, to try new things, throwing aside whatever is not good and adopting with zeal whatever is useful.

Dr. Spalding's address was received with applause, and upon its conclusion he retired from the chair, when his successor, Dr. Barron, was conducted to the chair by Drs. Forbes and French, and delivered a beautiful and chaste address, in which he alluded to the organization of the W. D. Society, and urged the necessity of dental associations, colleges and periodicals; exhorting his professional brethren to avail themselves of the advantages of the best means of instruction in the country—hinting that one poor operator will do more harm, than several good operators can do good or would be able to remedy. The happy manner in which Dr. B. alluded to the laborious and toilsome road to scientific preferment and eminence, was only excelled by his beautiful allusion to the facility and harmony with which the preceding officer retired and gave place to his successor, hoping that the same good feeling and good fellowship might prevail in our civil government.

Matters of a business character occupied the attention of the Association during the remainder of the morning session.

Adjourned till 2 o'clock, P. M.

2 o'clock, P. M.—Association met pursuant to adjournment. The afternoon session was occupied in the discussion of the following propositions:

The treatment of the roots of teeth in which the nerves have been destroyed and removed.

The effect of diseased shedding teeth upon the adjoining permanent teeth.

The causes and treatment of caries of the teeth.

Which was participated in by the members generally, with interest, and evinced much scientific research.

Saturday, July 7th, 8 o'clock, A. M.—The Society met pursuant to adjournment, President in the chair.

An appropriation of two hundred dollars was made, to be given to any Chemist who would discover or make a plastic compound that can be substituted for gold in filling teeth.

It was stated that there now is the amount of two million dollars of gold per year used for dental purposes.

The Society proceeded to discuss the sixth proposition, viz: the relative value of Gold, Platina, Hard Rubber, Silver, etc., as a base for artificial teeth. Gold and vulcanite were preferred. The advertising, cheap dentist, it was said, might invariably be set down as one unworthy of public confidence, as each practitioner might be supposed the best judge of his own services.

The subject of Professional Courtesy was introduced and discussed, embracing the following points, viz: Fraternal intercourse (beginning with who is my brother); treatment of neighboring practitioners; decorum to patrons; courtesy to physicians; courtesy to clergymen, etc.

The remarks on this subject were truly pleasant and edifying. Drs. Forbes, Spalding, Barron, Blake, Peebles and French gave their views upon the subject.

The remarks of Dr. Forbes on this subject were highly interesting, and expressed so fully the sentiments of the Association, that he was requested to prepare them for publication. Drs. Barron and Spalding were also requested to furnish their remarks for publication.

At 12 M., the Society adjourned to meet in St. Louis, in July, 1861.

On Friday evening the dentists of the city invited the members of the Faculty and a few citizens of other avocations, to an entertainment at the St. Nicholas Hotel. On entering the dining room, their eyes were greeted by one of the most magnificently spread tables that has ever been set forth in this city. The bill of fare, which, though quite enough to satisfy the most dainty palate, gives but a faint idea of the burden the tables bore, in the elegant taste displayed in the arrangement. There was a moistening and exhilarating fluid in tin topped bottles, passed freely around, and wit, sentiment and oratory entertained the company till a late hour. A decided healthy feeling prevailed before the ceremonies were ended, and each one felt that it was good to be there.

The proprietors of the St. Nicholas did themselves great credit on this occasion, and their house will long be remembered by their guests. Dr. Freeman's viands are decidedly better "to taste" than his medicines.

